# Anti-inflammatory effects of adenosine N1-oxide

**DOI:** 10.1186/s12950-014-0045-0

**Published:** 2015-01-20

**Authors:** Keizo Kohno, Emiko Ohashi, Osamu Sano, Hajime Kusano, Toshio Kunikata, Norie Arai, Toshiharu Hanaya, Toshio Kawata, Tomoyuki Nishimoto, Shigeharu Fukuda

**Affiliations:** Core Technology Division, Research and Development Center, Hayashibara Co., Ltd, Okayama, Japan; Functional Dye Division, Functional Dye Department, Hayashibara Co., Ltd, Okayama, Japan; Applied Technology Division, Research and Development Center, Hayashibara Co., Ltd, Okayama, Japan

**Keywords:** Adenosine, Anti-inflammatory effect, Pro-inflammatory cytokines, Adenosine receptor agonists, Endotoxin shock

## Abstract

**Background:**

Adenosine is a potent endogenous anti-inflammatory and immunoregulatory molecule. Despite its promise, adenosine’s extremely short half-life in blood limits its clinical application. Here, we examined adenosine N1-oxide (ANO), which is found in royal jelly. ANO is an oxidized product of adenosine at the N1 position of the adenine base moiety. We found that it is refractory to adenosine deaminase-mediated conversion to inosine. We further examined the anti-inflammatory activities of ANO *in vitro* and *in vivo*.

**Methods:**

The effect of ANO on pro-inflammatory cytokine secretion was examined in mouse peritoneal macrophages and the human monocytic cell line THP-1, and compared with that of adenosine, synthetic adenosine receptor (AR)-selective agonists and dipotassium glycyrrhizate (GK2). The anti-inflammatory activity of ANO *in vivo* was examined in an LPS-induced endotoxin shock model in mice.

**Results:**

ANO inhibited secretion of inflammatory mediators at much lower concentrations than adenosine and GK2 when used with peritoneal macrophages and THP-1 cells that were stimulated by LPS plus IFN-γ. The potent anti-inflammatory activity of ANO could not be solely accounted for by its refractoriness to adenosine deaminase. ANO was superior to the synthetic A1 AR-selective agonist, 2-chloro-N^6^-cyclopentyladenosine (CCPA), A2A AR-selective agonist, 2-[*p*-(2-carboxyethyl)phenethylamino]-5’-N-ethylcarboxamideadenosine hydrochloride (CGS21680), and A3 AR-selective agonist, N^6^-(3-iodobenzyl)adenosine-5’-N-methyluronamide (IB-MECA), in suppressing the secretion of a broad spectrum of pro-inflammatory cytokines by peritoneal macrophages. The capacities of ANO to inhibit pro-inflammatory cytokine production by THP-1 cells were comparable with those of CCPA and IB-MECA. Reflecting its potent anti-inflammatory effects *in vitro*, intravenous administration of ANO significantly reduced lethality of LPS-induced endotoxin shock. A significant increase in survival rate was also observed by oral administration of ANO. Mechanistic analysis suggested that the up-regulation of the anti-inflammatory transcription factor c-Fos was, at least in part, involved in the ANO-induced suppression of pro-inflammatory cytokine secretion.

**Conclusions:**

Our data suggest that ANO, a naturally occurring molecule that is structurally close to adenosine but is functionally more potent, presents potential strategies for the treatment of inflammatory disorders.

## Introduction

Acute local inflammation is a healthy immune response that protects the body from pathogens such as bacteria, viruses, fungi, and other parasites. Dendritic cells and macrophages encountering the pathogens are triggered to release cytokines and chemokines. In acute-phase inflammation, these cytokines and chemokines increase blood flow and vascular permeability along with the accumulation of fluid and leukocytes that are important for an effective defense [1]. However, in a variety of chronic pathological conditions, pro-inflammatory cytokines are released at high levels and mediate systemic inflammatory responses. These cytokines include IL-1, IL-6, IL-12, IL-23 and TNF-α. For instance, when TNF-α is released locally and physiologically at low levels, it plays beneficial roles in protective immune responses against infectious pathogens by helping neutrophil migration to a site of infection. However, when it is systemically released at high levels, TNF-α can cause severe inflammatory diseases. Clinical administrations of neutralizing antibodies against TNF-α or IL-6 have been successful in patients with rheumatoid arthritis, inflammatory bowel diseases and psoriasis [2-5].

Adenosine is a key molecule that regulates numerous physiological processes by activating four G-protein-coupled adenosine receptors (ARs), A1, A2A, A2B and A3 ARs. The nature and magnitude of the effect of adenosine on the cell depend on the extracellular adenosine concentrations, receptor density and the functional characteristics of the intracellular signaling pathways [6]. Adenosine is a potent endogenous anti-inflammatory and immunoregulatory molecule [6,7]. When adenosine is physiologically released from cells at sites of inflammation or tissue injury, it regulates the immune and inflammatory systems and plays a central role in wound healing by increasing angiogenesis through upregulating vascular endothelial growth factor [6]. It is well accepted that adenosine downregulates the release of pro-inflammatory mediators primarily through A2A AR [8,9]. Furthermore, adenosine mediates the anti-inflammatory effects of methotrexate [10]. Despite these beneficial properties, adenosine has an extremely short half-life because of its rapid metabolism in blood due to conversion by adenosine kinase to adenosine monophosphate (AMP) or its change to inosine by adenosine deaminase. These conversions prevent its clinical usage [7,11].

In this regard, the therapeutic benefit of AR-selective agonists is fully appreciated and varieties of selective agonists have been chemically synthesized. Using these synthetic adenosine agonists, clinical trials have been carried out, although some have been withdrawn due to potential side-effects or poor bioavailability [12,13].

In addition to the anti-inflammatory action through activation of A2A AR, the three remaining ARs possess some anti-inflammatory activity. For example, when an adenosine A1R agonist was injected 24 h prior to challenge with *E. coli*, upregulation of serum pro-inflammatory cytokines and peritoneal leukocyte recruitment were inhibited, thereby reducing the severity of peritonitis [14]. Moreover, A2B AR-deficient mice failed to induce regulatory T cells after endotoxin-induced pulmonary inflammation and underwent enhanced recruitment of pro-inflammatory effector T cells. Those results suggest that A2B AR serves a potent anti-inflammatory role through the upregulation of regulatory T cells [15]. It has been shown that methotrexate-induced adenosine downmodulates acute inflammation by activating A2A and/or A3 ARs [16]. These findings suggest that adenosine, as a physiological agonist, has therapeutic potential for inflammatory disorders through one or more of the ARs, if the drawbacks of short half-life could be overcome.

We have previously shown that royal jelly (RJ) contains low and high molecular weight substances (<5 kDa and >30 kDa, respectively) that can inhibit the secretion of pro-inflammatory cytokines by activated macrophages [17]. We previously isolated MRJP3 protein as a high molecular weight anti-inflammatory substance [17]. Using multiple steps of chromatography, we also isolated adenosine N1-Oxide (ANO), an anti-inflammatory substance from the low-molecular weight fraction of RJ. In this study, the anti-inflammatory actions of ANO were examined *in vitro* and *in vivo* and compared with those of adenosine, synthetic AR-selective agonists, and dipotassium glycyrrhizinate (GK2).

## Materials and methods

### Mice

BALB/c female mice, aged 8–12 weeks, were purchased from Charles River Japan (Kanagawa, Japan). All animal experiments described in this article were conducted according to the guidelines on Animal Experimentation at the R&D center of Hayashibara Co., LTD.

### Reagents

RJ that had been collected from Anhui in China was used. Lipopolysaccharide (LPS) (*E. coli* 055:B5), adenine, adenosine, zymosan A, Wortmannin and adenosine deaminase were purchased from Sigma-Aldrich Japan (Tokyo, Japan). Adenine N1-Oxide was purchased from MP Biomedicals (Santa Ana, CA). GK2 was purchased from Maruzen Pharmaceuticals (Hiroshima, Japan). Adenosine deaminase inhibitor, erythro-9-(2-hydroxy-3-nonyl) adenine hydrochloride (EHNA) was obtained from Enzo Life Sciences (Farmingdale, NY). Pam_3_CSK_4_ was purchased from Bachem (King of Prussia, PA). Poly I:C was purchased from Calbiochem (La Jolla, CA). Murine recombinant interferon-γ (IFN-γ), human TNF-α, and monoclonal antibodies (mAb) for human TNF-α ELISA were prepared and purified in our laboratories. Mouse cytokine standards for ELISA (TNF-α, IL-6, IL-10, and IL-12) were obtained from BD Pharmingen (San Diego, CA). The following pairs of mAbs for ELISA capture and biotinylated detection were purchased from BD Pharmingen: TNF-α, G281-2626 and MP6-XT3; IL-6, MP5-20 F3 and MP5-32C11; IL-10, JES5-2A5 and SXC-1; and, IL-12 p70, 9A5 and C17.8.

A1 AR-selective agonist 2-chloro-N^6^-cyclopentyladenosine (CCPA), A2A AR- selective agonist 2-[*p*-(2-carboxyethyl)phenethylamino]-5’-N-ethylcarboxamideadenosine hydrochloride (CGS21680), A3 AR-selective agonist N^6^-(3-iodobenzyl)adenosine-5’-N-methyluronamide (IB-MECA), and A3 AR-selective antagonist 3-ethyl-5-benzyl-2-methyl-4-phenylethynyl-6-phenyl-1,4-(±)-dihydropyridine-3, 5-dicarboxylate (MRS1911) and adenosine were purchased from Sigma-Aldrich Japan. A1 AR-selective antagonist 8-cyclopentyl-1,3- dipropylxanthine (DPCPX), A2A AR-selective antagonist 4-(2-[7-amino-2-(furan-2-yl)-[1,2,4]triazolo[1, 5-a][1,3,5]triazin-5-ylamino]ethyl) phenol (ZM241385), A2B AR-selective antagonist N-(4-cyanophenyl)-2-[4-(2, 3, 6, 7-tetrahydro-2, 6-dioxo-1, 3-dipropyl-1H-purin-8-yl)phenoxy]-acetamide (MRS1754) were purchased from Tocris Bioscience (Bristol, UK). N-[2-[[3-(4-Bromophenyl)-2-propenyl]amino]ethyl]-5-isoquinolinesulfonamide dihydrochloride (H-89) was purchased from Merck Millipore (Darmstadt, Germany).

### Isolation of ANO from extracts of RJ

Fresh RJ was suspended in 200 mM Tris–HCl buffer, pH 8.0. The supernatants of the RJ suspensions were collected by centrifugation at 10,000×g for 15 min at 4°C. The low-molecular weight fraction of RJ was prepared by centrifugation (2,000×g) using an Ultrafree centrifugal filter device, with a molecular cut-off of 6 kDa. From the low-molecular weight fraction of RJ, ANO was purified to homogeneity by sequential purification on three types of reversed-phase column chromatography: a Vydac 218TP510 column (Grace, Deerfield, IL), a TSKgel ODS-80Ts column (Tosoh, Tokyo, Japan), followed by an YMC-Pack ODA-A column (YMC, Kyoto, Japan). The yield of ANO was 123 ± 25 μg per 1 g of RJ (mean ± S.D., n = 3).

ANO structure was characterized spectroscopically by ^1^H and ^13^C NMR and electrospray ionization MS (ESI-MS): ESI-MS m/z 284.15 [M + H]^+^; ^1^H-NMR (dimethylsulphoxide-d_6_, 300 MHz) δ ppm: 8.663 (s, 1H), 8.565 (s, 1H), 7.5 - 9.0 (br, 2H), 5.885 (d, J = 5.4, 1H), 5.546 (d, J = 4.8, 1H), 5.240 (d, J = 3.3, 1H), 5.059 (bs, 1H), 4.530 (bd, J = 4.8, 1H), 4.148 (bd, J = 3.0, 1H), 3.946 (bd, J = 3.6, 1H), 3.670 (m, 1H), 3.553 (m, 1H); and ^13^C-NMR (dimethylsulphoxide-d_6_, 300 MHz) δ ppm: 143.28, 142.41, 148.32, 141.92, 118.82, 87.43, 85.49, 73.75, 70.13, 61.15. Chemical shifts in the ribose moiety of ANO were very close to those of adenosine in both ^1^H-NMR and ^13^C-NMR analyses. Chemical shifts in the adenine base moiety of ANO showed similar patterns as those of adenosine monophosphate N1-oxide (AMP N1-Oxide) reported previously [18].

### Synthesis of ANO

ANO was prepared according to the procedure described previously [19]. Adenosine (20 g) was suspended in 1 L of acetic acid, and 100 mL of 30% hydrogen peroxide solution was added to the suspension. The mixtures were stirred for 5 days at room temperature. After decomposing excess amounts of hydrogen peroxide by adding 5 g of 5% palladium on carbon (Kawaken Fine Chemicals, Tokyo, Japan) to the mixture, the palladium on carbon was separated by filtration. The filtrate was desiccated under reduced pressure. ANO crystallized after adding ethanol to the residue and was isolated by filtration. After repeated recrystallization from methanol, 8 g of ANO with 99.1% purity was obtained and used in this study.

### Cell cultures and stimulation

Murine peritoneal macrophages were elicited by intraperitoneal injection of 2 mL 4% Brewer’s thioglycollate medium (Nissui Pharmaceutical, Tokyo, Japan) into the peritoneal cavities of BALB/c mice. Peritoneal exudate cells were collected by lavage 3 to 4 days after injection. Cells were washed twice and plated in 10-cm diameter plastic dishes (Nippon Becton Dickinson, Tokyo, Japan) at a density of 1 × 10^8^ cells/dish in 10 mL of RPMI1640 medium (Nissui Pharmaceutical) containing 10% (v/v) FBS (Life Technologies, Grand Island, NY). After 2 h incubation at 37°C in a humidified atmosphere of 5% CO_2_ and 95% air, non-adherent cells were removed by rinsing. RPMI1640 medium containing 10% FBS was then added to the adherent cells that were recovered by scraping (Nippon Becton Dickinson). The recovered cells were used as macrophages. The murine macrophage-like cell line, RAW264.7 and the human monocytic cell line, THP-1 were maintained in RPMI1640 medium containing 10% FBS. THP-1 cells were cultured with 1 mM sodium butyrate for 4 days before being used in the experiments.

For pro-inflammatory cytokine production, peritoneal macrophages were seeded at 5 × 10^4^ cells per well in flat bottom 96-well microtiter plates and stimulated with LPS (1 μg/mL) with or without murine IFN-γ (muIFN-γ) (10 IU/mL) in the presence or absence of various concentrations of ANO, adenosine, adenine, adenine N1-oxide, or GK2 at 37°C for 24 h. In some experiments, peritoneal macrophages were stimulated with TLR agonists. Sodium butyrate-treated THP-1 cells (1 × 10^5^ cells per well) were stimulated with LPS (5 μg/mL) plus human IFN-γ (huIFN-γ) (500 IU/mL) (termed LPS/huIFN-γ). In other experiments, THP-1 cells were pretreated with AR-selective antagonists 30 min before stimulation with LPS/huIFN-γ in the presence or absence of 10 μM ANO. After 24 h, the culture supernatants were collected for the measurement of cytokines and PGE_2_. PGE_2_ was measured using an ELISA kit (Amersham Pharmacia Biotech, Tokyo, Japan). The lower limits of detection were 16, 50, 25, 250, 16 and 50 pg/mL for human TNF-α, murine TNF-α, IL-6, IL-10, IL-12 and PGE_2_, respectively.

For measurement of cell proliferation, 20 μL alamarBlue dye (Trek Diagnostic Systems, OH), a redox indicator, was added to each microplate well for the last 2 to 3 h of the incubation period. Fluorescence intensity (FI) was measured at 544 nm excitation wavelength and 590 nm emission wavelength.

### Stability of ANO in the presence of adenosine deaminase

The stability of ANO and adenosine in culture medium containing 10% FBS at 37°C was examined by quantifying the concentrations at the end points of the different incubation periods by reverse phase high pressure liquid chromatography (HPLC) using an ODS AQ-303 column (YMC). In some experiments, an inhibitor of adenosine deaminase, EHNA, was added together with adenosine. In separate experiments, ANO or adenosine was mixed with 6.7 U/L of adenosine deaminase enzyme in 53.3 mM potassium phosphate buffer containing 0.003% bovine serum albumin, and the mixtures were incubated for up to 60 min at 37°C. The time course of ANO or adenosine conversion was analysed by reverse phase HPLC.

### LPS-induced endotoxin shock studies

BALB/c mice were intravenously administered saline or ANO just before intraperitoneal injection of LPS (18 mg/kg) (n = 6 mice in each group). In a separate experiment, ANO was administered orally 1 h before and 1 and 6 h after LPS injection (n = 8 mice in each group). Survival of the mice was monitored for 3 days after LPS injection. In a parallel experiment, blood samples were collected from the abdominal aorta 2 h after the LPS injection. Sera from mice were obtained after centrifugation for 10 min at 4°C and stored at - 80°C until cytokine measurements were performed.

### RNA extraction and quantitative real-time polymerase chain reaction (PCR)

Briefly, RAW264.7 cells (1 × 10^6^/mL) were stimulated with LPS (2 μg/mL) in the presence or absence of 10 μM ANO in 6-well plates, and incubated for 0.5 to 3 h at 37°C in 5% CO_2_. Total RNA was extracted from RAW264.7 cells using an RNeasy Mini kit (QIAGEN, Tokyo, Japan) and DNase (QIAGEN) according to the manufacturer’s instructions. Subsequently, first-strand cDNA was synthesized using Superscript® VILO™ cDNA synthesis kit (Life Technologies, Carlsbad, CA). Specific primers for PCR analysis of *c-fos* were identical to those described previously [20]. PCR assay was carried out with the following sense and antisense primers: *c-fos* [GenBank: NM_010234], CGAAGGGAACGGAATAAGAT and GCAACGCAGACTTCTCATC; *Gapdh* [GenBank: NM_008084], ACCATCTTCCAGGAGCGAG and AGTGATGGCATGGACTGTGG. Synthesized cDNA was mixed with SYBR Green Master Mix (Roche, Mannheim, Germany) and different sets of gene-specific primers. Real-time PCR was performed using a LightCycler 480 system (Roche). For quantitative purposes, the expression of the *c-fos* gene was normalized to a house keeping gene, *Gapdh*.

### Western immunoblot analysis of cell signaling molecules

RAW264.7 cells were stimulated with LPS (2 μg/mL) in the presence or absence of various concentrations of ANO for 30 min at 37°C. Cellular proteins were prepared in lysis buffer (20 mM Tris–HCl, pH 7.5 containing 0.15 M NaCl, 1 mM EDTA, 0.1% SDS, 1% Triton X-100, 0.5% sodium deoxycholate and inhibitors of proteases and phosphatases). Nuclei and cell debris were removed by centrifugation at 500 g for 10 min. Cell lysates (20 μg) were separated by sodium dodecyl sulfate-polyacrylamide gel electrophoresis (SDS-PAGE) (Multigel 10/20; COSMO Bio, Tokyo, Japan) under reducing conditions, and transferred to polyvinylidene difluoride (PVDF) membranes (Merck Millipore Japan, Tokyo, Japan) by electrophoretic transfer. PVDF membranes were then blocked with a solution containing 10% Block Ace (Dainippon Pharmaceutical, Osaka, Japan) for 0.5 h, and incubated with anti-c-Fos mAb (9 F6; Cell Signaling Technology, Danvers, MA) for 1 h. After washing the primary mAb, membranes were washed three times with Tris-buffered saline containing 0.05% Tween 20, and then incubated with a 1:1000 dilution of secondary Ab conjugated to horseradish peroxidase (Dako) for 1 h. The membranes were then washed three times and reaction products visualized using the enhanced chemiluminescence Western blot system (Amersham). The same membrane was stripped and reprobed with anti-c-Jun mAb (60A8; Cell Signaling Technology) and anti-β-actin (ACTBD11B7; Santa Cruz Biotechnology, Santa Cruz, CA), and then subjected to Western blotting analysis as described above.

### Statistical analysis

Statistical analysis of the data was performed by Dunnett’s test as appropriate. Survival differences were evaluated with the log-rank tests using Kaplan-Meier survival curves. *P*-values < 0.05 were considered statistically significant.

## Results

### ANO inhibited IL-6 secretion by macrophages in response to pathogen-associated molecular patterns

**Figure 1:**
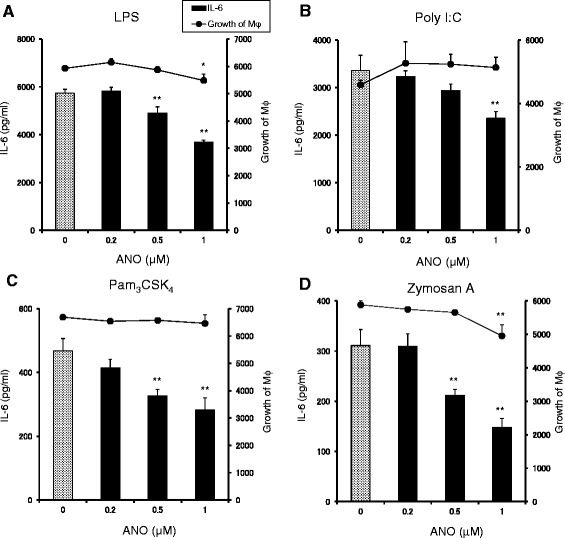
**ANO inhibited IL-6 production by peritoneal macrophages stimulated with TLR agonists.** Peritoneal macrophages (5 × 10^4^/well) were stimulated with LPS (2 μg/mL) **(A)**, Poly I:C (50 μg/mL) **(B)**, Pam_3_CSK_4_ (5 μg/mL) **(C)** and zymosan A (100 μg/mL) **(D)** in the presence or absence of various concentrations of ANO at 37°C for 24 h. Levels of IL-6 in the culture supernatants were determined by ELISA. Growth of macrophages was assessed by adding 20 μl/well of alamarBlue™ dye for the last 2 to 3 h of the incubation period and expressed as FI values. Values represent the means ± S.D. of triplicate cultures. Results are representative of two separate experiments with similar results. *p < 0.05; **p < 0.01, significantly different when compared with control culture.

During our screening program to identify substance(s) that inhibited secretion of pro-inflammatory cytokines, we isolated ANO from the low-molecular weight fraction of RJ. We then examined the effects of ANO on pro-inflammatory cytokine production by macrophages activated by their recognition of pathogen-associated molecular patterns (PAMPs) via Toll-like receptors (TLRs). Towards that end, the following activators were used: LPS (TLR4 agonist), poly I:C (TLR3 agonist), Pam_3_CSK_4_ (TLR 1/2 agonist) or zymosan A (TLR2 agonist). Exposure of murine peritoneal macrophages to these PAMPs induced secretion of substantial amounts of IL-6. When ANO was added to the culture in the presence of the bacterial and viral PAMPs, IL-6 secretion was significantly inhibited in a dose-dependent fashion (Figure 1). Since the macrophage growth curve was not parallel to the IL-6 inhibition curve, the inhibition was not due to decreases in macrophage growth (Figure 1).

### Anti-inflammatory effects of ANO are superior to those of adenosine and dipotassium glycyrrhizate

**Figure 2:**
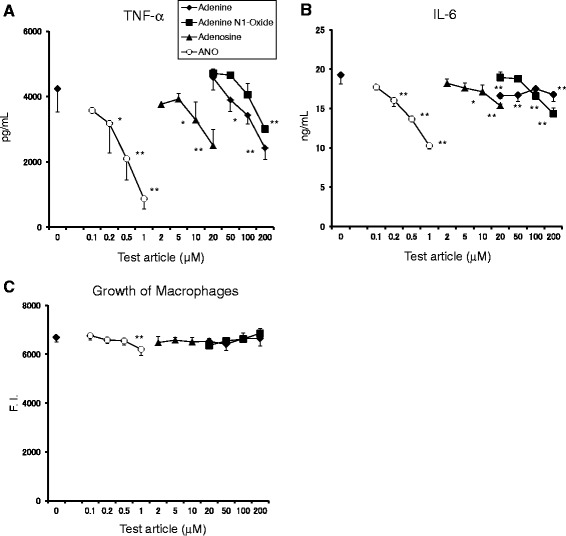
**Inhibitory effects of ANO, adenosine, adenine and adenine N1-oxide on TNF-α and IL-6 secretion by LPS/muIFN-γ-stimulated peritoneal macrophages.** Peritoneal macrophages (5 × 10^4^/well) were stimulated with LPS (1 μg/mL) and muIFN-γ (10 IU/mL) in the presence or absence of various concentrations of ANO, adenosine, adenine and adenine N1-oxide at 37°C for 24 h. Levels of TNF-α **(A)** and IL-6 **(B)** in the culture supernatants and growth of macrophages **(C)** were determined as described in Figure 1. Values represent the means ± S.D. of quadruplicate cultures. Results are representative of two separate experiments with similar results. *p < 0.05; **p < 0.01, significantly different when compared with control culture.

ANO is an oxidized product of adenosine at the N1 position of the adenine moiety. To explore the relationship between structure and activity, we compared the inhibitory effects of ANO on pro-inflammatory cytokine production with those of adenosine, adenine and adenine N1-oxide. For this purpose, murine peritoneal macrophages were stimulated with 1 μg/mL LPS plus 10 IU/mL murine IFN-γ (LPS/muIFN-γ) to induce multiple pro-inflammatory mediators. IFN-γ primes macrophages, enhancing their response to LPS [21]. Moreover, IFN-γ is a critical mediator of endotoxin hypersensitivity induced by bacteria [22]. Figure 2A and B show the dose–response curves of the inhibitory effects of ANO and related compounds on the release of TNF-α and IL-6. ANO efficiently inhibited both TNF-α and IL-6 production in a dose-dependent fashion. Although minimal inhibition of macrophage growth was observed at 1 μM ANO, significant reductions in the production of both TNF-α and IL-6 were observed at concentrations that had no effect on macrophage growth. Therefore, it seems likely that inhibition of pro-inflammatory cytokines by ANO was not due to a reduction of cell proliferation (Figure 2C). Adenosine also inhibited the secretion of both TNF-α and IL-6 in a dose-dependent manner, but much higher concentrations were required to achieve reductions observed with ANO (Figure 2A and B). Unexpectedly, adenine significantly inhibited the release of TNF-α at 10 times higher concentrations than adenosine. IL-6 production was also significantly inhibited by adenine, while no clear dose–response effect was observed. Addition of adenine N1-oxide also resulted in reduction of both TNF-α and IL-6. However, the inhibitory action of adenine N1-oxide was comparable or inferior to that of adenine (Figure 2A and B).

**Figure 3:**
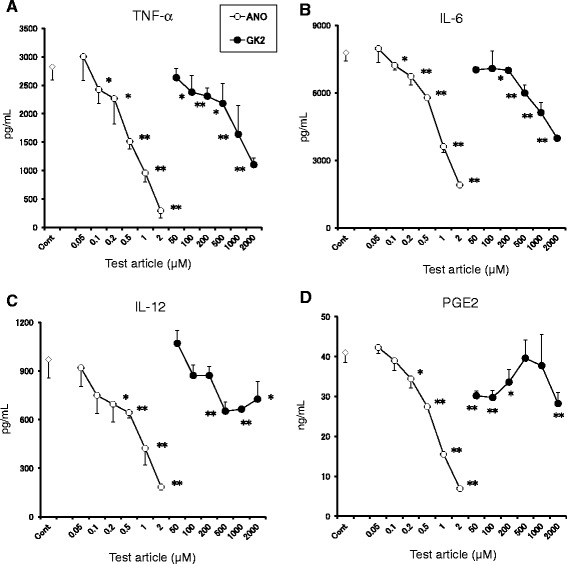
**Inhibitory effects of ANO and GK2 on the release of inflammatory mediators by LPS/muIFN-γ-stimulated peritoneal macrophages.** Peritoneal macrophages (5 × 10^4^/well) were stimulated with LPS (1 μg/mL) and muIFN-γ (10 IU/mL) in the presence or absence of various concentrations of ANO and GK2 at 37°C for 24 h. Levels of TNF-α **(A)**, IL-6 **(B)**, IL-12 **(C)** and PGE2 **(D)** in the culture supernatants were determined by ELISA. Values represent the means ± S.D. of triplicate cultures. Results are representative of two separate experiments with similar results. *p < 0.05; **p < 0.01, significantly different when compared with control culture.

Figure 3 compares the inhibitory effects of ANO and GK2 (a broadly used anti-inflammatory drug) on the release of multiple pro-inflammatory mediators such as TNF-α, IL-6, IL-12 and prostaglandin E2 (PGE2) by peritoneal macrophages in response to LPS/muIFN-γ. ANO efficiently reduced the release of these pro-inflammatory molecules in a dose-dependent fashion. Inhibition of pro-inflammatory mediators was also observed with GK2, although to a much lesser extent than ANO. In accordance with a previous report [23], GK2 had a bimodal effect on PGE2 production.

In addition, ANO exhibited more potent inhibitory effects than adenosine and GK2 on the secretion of both TNF-α and IL-6 by LPS/huIFN-γ-stimulated THP-1 cells. The EC50s for ANO-, adenosine-, and GK2-induced inhibition of TNF-α secretion were 4.9 μM, 55 μM, and 910 μM, respectively. The EC50s for ANO-, adenosine-, and GK2-induced inhibition of IL-6 secretion were 4.9 μM, 40 μM, and 1,000 μM, respectively.

### ANO is refractory to deamination by adenosine deaminase

**Figure 4:**
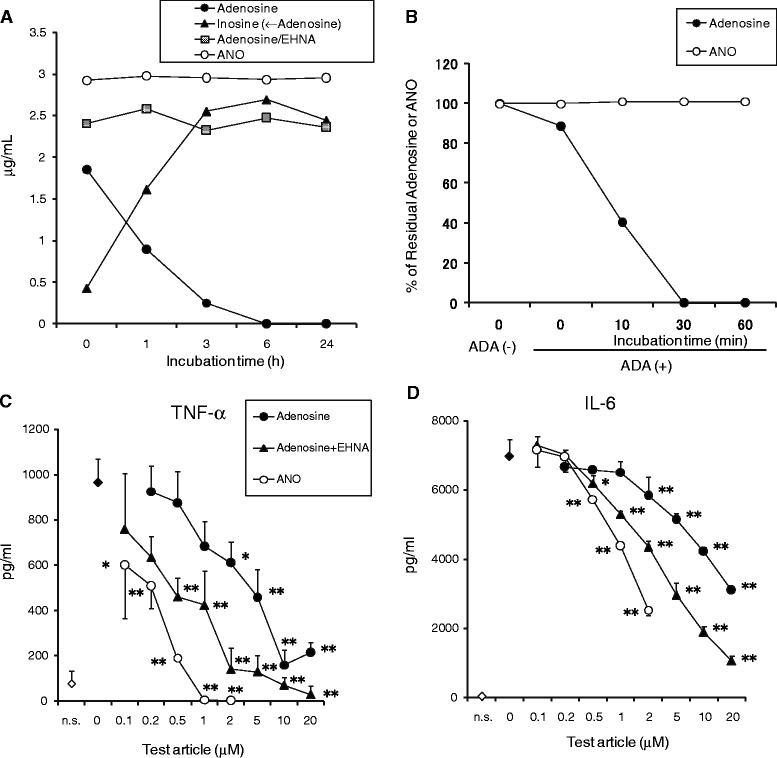
**ANO was refractory to deamination by adenosine deaminase.** Stability of 10 μM ANO or adenosine in RMPM 1640 medium supplemented with 10% FBS was assessed in the presence or absence of EHNA (10 μM) for up to 24 h at 37°C. Concentrations of ANO, adenosine or inosine at selected time points were quantified by HPLC **(A)**. Reactivity of 10 μM ANO or adenosine with 6.7 U/L of adenosine deaminase enzyme was assessed for up to 60 min at 37°C in 53.3 mM potassium phosphate buffer containing 0.003% bovine serum albumin. Data are expressed as percentages of residual amounts of ANO or adenosine at each point in time **(B)**. Anti-inflammatory effects of adenosine in the presence or absence of EHNA (10 μM) on TNF-α and IL-6 production by LPS (2 μg/mL)-stimulated peritoneal macrophages were compared with those of ANO. Levels of TNF-α and IL-6 in the culture supernatants were determined by ELISA after incubation for 24 h at 37°C **(C and D)**. Results are representative of three separate experiments with similar results (A and B). In (C) and (D), values represent the means ± S.D. of triplicate cultures and are representative of two separate experiments with similar results. Inosine (←Adenosine) denotes amounts of inosine converted from 10 μM adenosine by adenosine deaminase. *p < 0.05; **p < 0.01, significantly different when compared with control culture.

Adenosine deaminase irreversibly converts adenosine to inosine. Since adenosine deaminase is present in serum, the stability of ANO and adenosine in culture medium supplemented with 10% FBS was examined. As shown in Figure 4A, 10 μM ANO was stable over a period of 24 h, whereas the same concentration of adenosine was rapidly degraded and was not found in the medium at the end of a 6 h incubation period. Conversely, the levels of inosine in the culture medium increased. When 10 μM adenosine was incubated in the presence of the adenosine deaminase inhibitor, EHNA (10 μM), degradation of adenosine was not observed (Figure 4A). We then compared the reactivities of ANO and adenosine to adenosine deaminase (Figure 4B). Whereas adenosine rapidly disappeared after incubation with adenosine deaminase, no change in ANO content was observed during 60 min incubation, suggesting that ANO is refractory to deamination by adenosine deaminase.

Next, we examined whether the difference in anti-inflammatory activities *in vitro* between ANO and adenosine could be ascribed to the refractoriness to adenosine deaminase. For this purpose, peritoneal macrophages were stimulated with LPS in the presence or absence of various concentrations of ANO or adenosine with or without 10 μM EHNA. As shown in Figure 4C and D, the inhibitory activity of adenosine on both TNF-α and IL-6 secretion was increased by the addition of EHNA but was still inferior to that of ANO.

### Comparison of ANO and AR-selective agonists for their capacities to inhibit pro-inflammatory cytokine production

**Figure 5:**
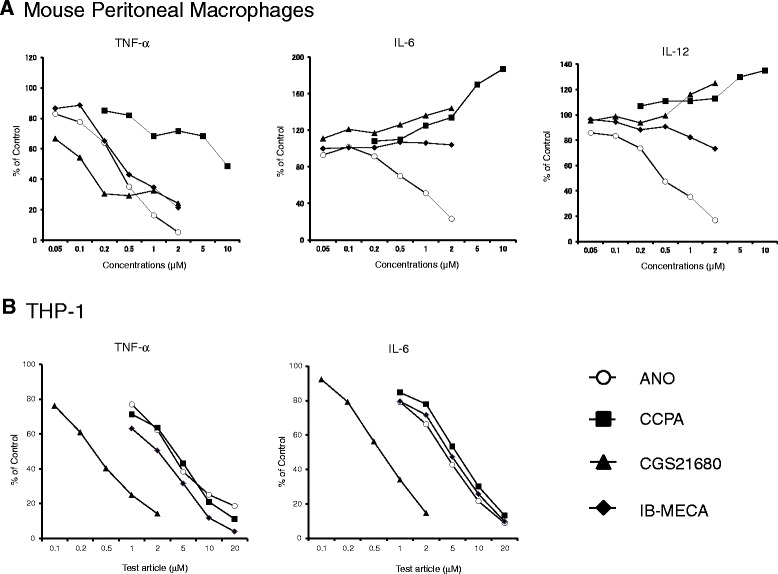
**Comparison of anti-inflammatory activities of ANO and AR-selective agonists.** Peritoneal macrophages (5 × 10^4^/well) were stimulated with LPS (1 μg/mL) and muIFN-γ (10 IU/mL) in the presence or absence of various concentrations of ANO or A1 AR-, A2A AR- and A3 AR-selective agonists (CCPA, CGS21680 and IB-MECA, respectively) at 37°C for 24 h **(A)**. Sodium butyrate-treated THP-1 cells (1 × 10^5^ /well) were stimulated with LPS (5 μg/mL) plus huIFN-γ (500 IU/mL) in the presence or absence of various concentrations of ANO or AR-selective agonists at 37°C for 24 h **(B)**. Levels of TNF-α, IL-6 and IL-12 in the culture supernatants were determined by ELISA. Values represent the percentage of control culture. Results are representative of two separate experiments with similar results.

A large number of AR-selective agonists have been chemically synthesized, some of which are under evaluation in clinical trials [13]. We compared the inhibitory potencies of ANO and three AR-selective agonists (CCPA for A1 AR agonist, CGS21680 for A2A AR agonist, and IB-MECA for A3 AR agonist). For this purpose, murine peritoneal macrophages were stimulated for 24 h with LPS/muIFN-γ in the presence or absence of ANO or the three agonists, and pro-inflammatory cytokines secreted into culture medium were determined. A2B AR-selective agonist with an adenosine-like structure is currently unavailable. As shown in Figure 5A, ANO and three AR-selective agonists all inhibited TNF-α secretion in a dose-dependent manner. Among the four agents, CGS21680 was most effective, especially at concentrations below 0.5 μM. TNF-α inhibitory potencies of ANO and IB-MECA were comparable. CCPA was less effective, but significantly inhibited TNF-α production, although CCPA is an A1 AR-selective agonist. In particular, ANO efficiently inhibited the release of both IL-6 and IL-12 production (Figure 5A). Interestingly, both CCPA and CGS21680 increased rather than reduced the release of IL-6 and IL-12. IB-MECA exhibited a slight reduction in IL-12 secretion, but it had no effect on IL-6 secretion.

The synthetic agonists displayed different reactivities to human THP-1 cells. ANO and the three agonists all inhibited the release of both TNF-α and IL-6 by THP-1 cells in response to LPS/huIFN-γ (Figure 5B). CGS21680 was most efficient. The inhibitory action of ANO was comparable to those of the other two agonists, CCPA and IB-MECA. The inhibitory effects by these agents did not appear to be caused by toxicity to THP-1 cells because there were no significant changes in cell viability or cell numbers at the concentrations employed as evaluated by trypan blue dye exclusion (data not shown).

### Examination of the type of AR that was involved in the anti-inflammatory actions of ANO

**Figure 6:**
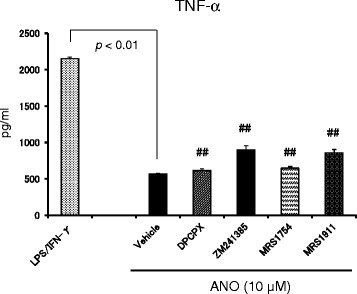
**Examination of the type of AR involved in the anti-inflammatory activity of ANO.** Sodium butyrate-treated THP-1 cells (1 × 10^5^ /well) were pretreated with 2 μM of A1 AR-, A2A AR-, A2B AR- and A3 AR-selective antagonists (DPCPX, ZM241385, MRS1754 and MRS1911, respectively) for 30 min before stimulation with LPS plus huIFN-γ in the presence of 10 μM ANO. After 24 h, levels of TNF-α in the culture supernatants were determined by ELISA. Values represent the means ± S.D. of triplicate cultures. Results are representative of two separate experiments with similar results. ##, *p* < 0.01, significantly different when compared with vehicle control culture in the presence of ANO.

We examined which type of AR was involved in ANO-mediated inhibition of pro-inflammatory cytokine production, first by using murine peritoneal macrophages. An A2A AR-selective antagonist (ZM241385) was unable to abrogate ANO-mediated inhibitory effect, whereas 68% - 82% of recovery of adenosine-mediated TNF-α inhibition was observed with ZM241385 (data not shown). We then used a human monocytic cell line, THP-1, which had been pretreated with 1 mM sodium butyrate for 4 days to increase the response to 5 μg/mL LPS plus 500 IU/mL human IFN-γ (LPS/huIFN-γ) [24]. When sodium butyrate-pretreated THP-1 cells were stimulated with LPS/huIFN-γ, substantial amounts of TNF-α (2.1 ng/mL) were detected in the culture medium (Figure 6). Stimulation of the THP-1 cells with LPS/huIFN-γ in the presence of 10 μM ANO resulted in a 73.6% decrease of TNF-α secretion without inhibiting cell growth. Specifically, the number of cells per well averaged 1.36 ± 0.15 × 10^5^ cells in the LPS/huIFN-γ culture vs. 1.39 ± 0.11 × 10^5^ cells in the LPS/huIFN-γ plus 10 μM ANO culture. When THP-1 cells were pretreated with 2 μM AR antagonists for 0.5 h prior to stimulation with LPS/huIFN-γ in the presence of 10 μM ANO, slight but significant recoveries of TNF-α inhibition were observed with ZM241385 and MRS1911 (an A3 AR antagonist) by 21% and 18%, respectively. Minimal but significant recoveries of TNF-α inhibition were also observed with MRS1754 (an A2B AR antagonist) and DPCPX (an A1 AR antagonist) by 12% and 6%, respectively. Increasing the concentrations of AR antagonists to 5 μM gave similar results (data not shown).

### ANO reduced the lethality caused by LPS-induced endotoxin shock

**Figure 7:**
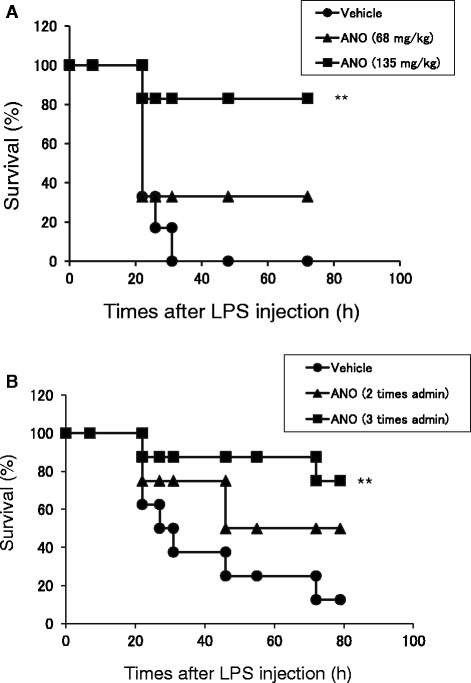
**LPS-induced endotoxin shock.** BALB/c mice were intravenously administered saline or ANO (68 mg/kg or 135 mg/kg) immediately before intraperitoneal injection with LPS (18 mg/kg) (n = 6 mice in each group) **(A)**. In a separate experiment, ANO (200 mg/kg) was administered orally 1 h before and 1 and 6 h after LPS injection (n = 8 mice in each group) **(B)**. Survival of mice was monitored for 3 days after LPS injection. Results are representative of two separate experiments with similar results. **p < 0.01, significantly different when compared with vehicle control.

Since ANO efficiently reduced pro-inflammatory cytokine secretion by LPS/muIFN-γ-stimulated macrophages, we examined the anti-inflammatory actions of ANO in LPS-induced endotoxin shock. When LPS was injected intra-peritoneally (18 mg/kg) immediately after intravenous administration of vehicle (PBS), all the mice died within 27 h (Figure 7A). In contrast, intravenous administration of ANO (68 mg/kg and 135 mg/kg) immediately before LPS injection reduced lethality in a dose-dependent manner. Significant prolongation of survival was observed upon administration of 135 mg/kg ANO, and 5 out of 6 mice were alive 3 days after LPS administration. We also examined the effect of oral administration of ANO on the survival of endotoxemic mice (Figure 7B). When 200 mg/kg of ANO was orally administered three times (1 h before and 1 and 6 h after LPS injection), significant prolongation of survival was observed and 6 out of 8 mice were alive after 3 days, whereas 7 out of 8 mice died if treated with vehicle alone. Oral administration of ANO 1 h before and 1 h after LPS injection resulted in increased survival rates (Figure 7B), although the data did not achieve statistical significance (*p* = 0.11).

**Figure 8:**
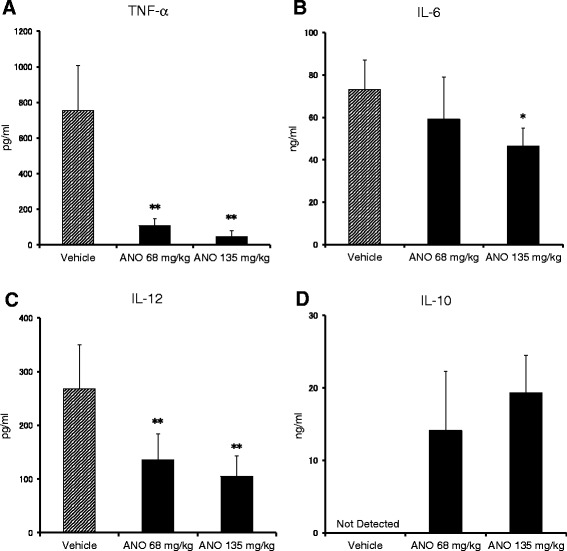
**Levels of pro-inflammatory and anti-inflammatory cytokines in the sera of endotoxemic mice.** ANO was administered intravenously as described in Figure 7A. Blood samples were collected from the abdominal aorta 2 h after LPS injection. Serum levels of TNF-α **(A)**, IL-6 **(B)**, IL-12 **(C)** and IL-10 **(D)** were measured by ELISA. Values represent the means ± S.D. of six mice in each group. Results are representative of two separate experiments with similar results. *p < 0.05; **p < 0.01, significantly different when compared with vehicle control.

Since excessive production of pro-inflammatory cytokines plays a critical role in animal models of septic shock [25], we measured serum levels of pro-inflammatory and anti-inflammatory cytokines 2 h after LPS administration. In accordance with prolongation of survival, serum levels of pro-inflammatory cytokines were significantly decreased in a dose-dependent manner by intravenous ANO administration (Figure 8A - C). In particular, serum TNF-α levels were decreased by 94% at 135 mg/kg of ANO administration (Figure 8A). With regard to anti-inflammatory cytokine IL-10, it was below detection in the sera of mice injected intravenously with PBS followed by intraperitoneal LPS injection (Figure 8D). However, when ANO was administered intravenously just before LPS injection, serum IL-10 levels were up-regulated and 14–19 ng/mL of IL-10 were detected.

### ANO and adenosine differentially inhibited TNF-α secretion by LPS-stimulated peritoneal macrophages

**Figure 9:**
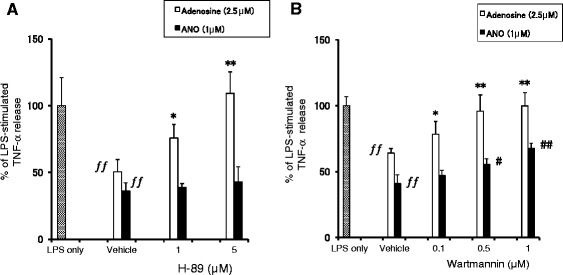
**Effect of H-89 or Wartmannin on ANO-induced inhibition of TNF-α production.** Peritoneal macrophages were incubated with various concentrations of H-89 **(A)** or Wartmannin **(B)** for 30 min before stimulation with LPS (2 μg/mL) in the presence of absence of ANO (1 μM) or adenosine (2.5 μM). After 24 h, levels of TNF-α in the culture supernatants were determined by ELISA. Values represent the means ± S.D. of quadruplicate cultures. Results are expressed as the percentage of TNF-α released from macrophages in response to LPS and are representative of two separate experiments with similar results. ƒƒ, *p* < 0.01, significantly different when compared with LPS stimulation in the absence of adenosine or ANO. *p < 0.05; **p < 0.01, significantly different when compared with vehicle control culture in the presence of adenosine. #, *p* < 0.05; ##, *p* < 0.01, significantly different when compared with vehicle control culture in the presence of ANO.

We found that ANO inhibited TNF-α secretion by LPS/huIFN-γ-stimulated THP-1 cells partially through A2A AR (Figure 7). Classical signaling through A2A AR depends on its coupling to the heterotrimeric Gs protein and stimulation of adenylyl cyclase, resulting in elevation of intracellular levels of cyclic AMP (cAMP) [6-8]. We observed that ANO increased cAMP levels in both peritoneal macrophages and a murine macrophage cell line, RAW264.7 cells (data not shown). Elevation of cAMP in RAW264.7 cells inhibited LPS-induced TNF-α production via the protein kinase A (PKA)-dependent pathway [26]. Therefore, we examined the effect of a PKA inhibitor (H-89) on adenosine- or ANO-induced inhibition of TNF-α secretion by LPS-stimulated peritoneal macrophages. In this experiment, to exclude extraneous signaling events exerted by muIFN-γ, macrophages were stimulated with LPS only. As shown in Figure 9A, adenosine-induced inhibition of TNF-α secretion was recovered in a dose-dependent manner by pre-incubation with H-89, and complete recovery was observed with 5 μM H-89. However, H-89 had no effect on the ANO-induced inhibition of TNF-α secretion.

c-Fos protein induced by a Wortmannin-sensitive kinase is responsible for the pro-inflammatory cytokine-suppressive action of cAMP [27]. Peritoneal macrophages were pretreated with Wartmannin before stimulation with LPS in the presence of adenosine or ANO. Adenosine-induced inhibition of TNF-α secretion was restored by Wartmannin pretreatment in a dose-dependent manner and complete recovery was observed at 0.5 μM Wartmannin (Figure 9B). Pretreatment with Wartmannin also significantly restored the ANO-induced inhibition of TNF-α secretion in a dose-dependent fashion, although the recovery was not complete.

**Figure 10:**
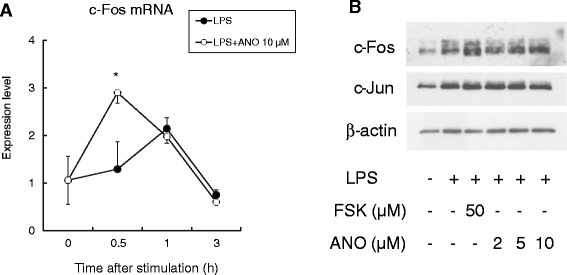
**Effect of ANO on mRNA and protein expression of c-Fos in LPS-stimulated RAW264.7 cells.** RAW264.7 cells were treated with LPS (2 μg/mL) and ANO (10 μM) for the indicated times. *c-fos* mRNA was analyzed by real-time PCR **(A)**. c-Fos and c-Jun proteins were assessed by immunoblotting **(B)**.

We next examined the effect of ANO on mRNA and protein expression of c-Fos in LPS-stimulated RAW264.7 cells. As shown in Figure 10A, the *c-fos* mRNA expression significantly increased 0.5 h after LPS stimulation in the presence of 10 μM ANO compared with stimulation by LPS alone. Expression of c-Fos protein was analyzed by Western blotting. Because of multiple phosphorylation sites, c-Fos was detected as multiple bands on SDS-PAGE with different molecular weights [27]. As shown in Figure 10B, ANO treatment caused a dose-dependent increase in c-Fos protein expression 1 h after LPS stimulation. c-Fos together with Jun family proteins constitutes the dimeric transcription factor AP-1. However, ANO had no effect on the expression of c-Jun protein.

## Discussion

In this study, we have shown that ANO is contained in RJ, which has been widely consumed as a dietary supplement and shows anti-inflammatory actions *in vitro* and *in vivo* [17,28]. RJ contains AMP and its N1-oxide, AMP N1-Oxide, both of which suppressed proliferation of rat pheochromocytoma PC12 cells and stimulated expression of neurofilament M [18]. It was also reported that RJ contains adenosine [29]. Using two-dimensional gel electrophoresis, we previously showed that glucose oxidase is present in RJ [30]. It is well known that glucose oxidase in honey oxidizes glucose to gluconic acid and hydrogen peroxide, the latter of which acts as an anti-microbial agent [31]. Therefore, it seems likely that ANO and AMP N1-Oxide in RJ are oxidative products of adenosine and AMP, respectively, by hydrogen peroxide generated by glucose oxidase.

In peritoneal macrophages, LPS/muIFN-γ upregulated the production of both TNF-α and IL-6. This process was significantly inhibited by adenine at concentrations ≥10 times of adenosine (Figure 2A and B), although the mechanism of action is unclear. Adenine N1-oxide also significantly reduced pro-inflammatory cytokines. However, the inhibitory action of adenine N1-oxide was comparable or inferior to that of adenine. This outcome contrasts with the superiority of ANO to adenosine in anti-inflammatory functions. These results suggest that the ribose moiety plays an essential role for manifestation of potent inhibitory activity in ANO.

ANO inhibited IL-6 secretion by peritoneal macrophages stimulated by TLR 1/2, TLR 3 and TLR 4 agonists (Figure 1). The effect of AR-selective agonists on cytokine production by human mononuclear cells depends on the TLR subtype [32]. For instance, CGS 21680 inhibited TLR 4-mediated TNF-α release but potentiated TLR 3- and TLR 5-mediated IL-6 release. In our study, we observed that the effects of AR-selective agonists on cytokine production were different depending on the pro-inflammatory cytokines released even under the same stimulatory conditions. Furthermore, functional differences between species in the anti-inflammatory actions of these agonists were observed. Both CCPA and CGS21680 significantly inhibited TNF-α secretion but potentiated IL-6 and IL-12 secretion by peritoneal macrophages stimulated with LPS/muIFN-γ, while these two agonists inhibited both TNF-α and IL-6 secretion by LPS/huIFN-γ-stimulated THP-1 cells (Figure 5). However, ANO efficiently inhibited all of those pro-inflammatory cytokines under the same stimulatory conditions. These results suggest that the anti-inflammatory effects of ANO are not restricted to a particular pro-inflammatory cytokine or to a situation in which a specific pathogen was involved.

At present, the differences in the response of the two agonists (CCPA and CGS21680) to mouse and human monocytes/macrophages remain unclear. It has been shown that the signaling pathways used by the A2A receptor may vary with the cellular background and the signaling machinery that the cell possesses [33]. Furthermore, species differences in the order of potency of A2A AR agonists have been reported [34]. These findings may provide insight for understanding the differential responses observed in our study.

We examined the role of ARs in ANO-mediated inhibition of TNF-α secretion by LPS/huIFN-γ-stimulated THP-1 cells. We found evidence for the involvement of all four ARs, although the extent of recoveries of TNF-α inhibition was different among the four AR-selective antagonists. Although still controversial, there is evidence that the four ARs can be effective in the treatment of inflammatory disorders, including ischemia-reperfusion injuries, endotoxin-induced injuries, arthritis and colitis. The protective effects of A1 AR have been demonstrated in models of ischemia reperfusion- and endotoxin-induced lung injury [35,36]. Due to its pre-dominant expression in immune cells and immunoregulatory actions, A2A AR has been shown to have high potential in the treatment of ischemia-, immune-, and inflammation-induced tissue injury [8,9]. Although it remains controversial whether A2B AR is pro-inflammatory or anti-inflammatory, recent studies using A2B AR-deficient mice showed that A2B AR played therapeutic roles in endotoxin-induced lung injury [15,37]. A3 AR activation inhibited of TNF-α release by endotoxin-stimulated monocyte/macrophage lineage cells [38] in accordance with our findings using IB-MECA.

A common feature of the above results suggests that activation of the four ARs might exert anti-inflammatory effects in TLR 4-mediated responses. In fact, CCPA, CGS21680 and IB-MECA significantly inhibited TNF-α production by LPS/IFN-γ-stimulated peritoneal macrophages and THP-1 cells (Figure 5). A2B AR-selective agonist with an adenosine-like structure was not available in our study. It is therefore not surprising that ANO-mediated inhibition of TNF-α release was slightly but significantly recovered by the four AR-selective antagonists. However, since the activation of A2B AR and A3 AR elicits a pro-inflammatory response in non-monocyte/macrophage lineage cells [39,40], this issue has to be taken into account for clinical application of ANO.

As expected from the *in vitro* results, intravenous ANO administration significantly reduced the lethality of LPS-induced endotoxin shock (Figure 7A). In marked contrast and as reported previously [41], adenosine failed to protect mice against endotoxin-induced mortality in our study, which is probably due to its rapid metabolism *in vivo* (data not shown). Reduced lethality in mice was also observed when ANO was administered orally before and after LPS injection (Figure 7B), suggesting that ANO could exert its anti-inflammatory effects systemically through oral administration.

In the LPS-induced endotoxin shock model, serum levels of pro-inflammatory cytokines (TNF-α, IL-6 and IL-12) were reduced but the levels of anti-inflammatory cytokine were upregulated by intravenous administrations of ANO (Figure 8). In a manner similar to that observed by intravenous administration, oral administration of ANO (200 mg/kg) 1 h before intraperitoneal injection of LPS (18 mg/kg) downregulated serum levels of TNF-α and IL-6 and upregulated IL-10 levels in serum when determined 2 h after LPS injection (data not shown). Pro-inflammatory cytokines TNF-α, IL-1 and IL-12, which are derived from monocyte/macrophages, play a key role in the pathogenesis of endotoxin shock [25]. In turn, anti-inflammatory cytokine IL-10 plays a protective role by inhibiting pro-inflammatory cytokines in LPS-induced endotoxin shock [42]. Therefore, it is most likely that the decrease in pro-inflammatory cytokines and upregulation of IL-10 explain the reduced lethality achieved by ANO injection.

The difference in the doses of ANO between *in vitro* and *in vivo* studies requires discussion. IB-MECA prevents lethality in endotoxemic mice when injected at 0.5 mg/kg 30 min before administration of a lethal dose of LPS [43]. In our study, a dose of 135 mg/kg of ANO was necessary to significantly prolong survival in endotoxemic mice, although ANO was comparable to or more potent than IB-MECA in suppressing the release of pro-inflammatory cytokines *in vitro* (Figure 5). ANO was refractory to adenosine-mediated conversion to inosine and therefore was stable in mouse serum (Figure 4 and data not shown). However, serum levels of ANO gradually decreased when ANO was incubated *in vitro* in blood taken from normal mice (data not shown). When ANO was added to the blood taken from mice injected with adenosine transport blocker dipyridamole, the decrease in serum levels of ANO was significantly inhibited (data not shown). These results suggest that ANO might be taken up through an adenosine transporter. The uptake of ANO through an adenosine transporter might be one reason why high-dose ANO was necessary in *in vivo* experiments. Structural modification of ANO to prevent its uptake by an adenosine transporter might decrease the effective dose *in vivo*. Studies are ongoing to address this issue.

Although ANO and adenosine are structurally similar, ANO’s capacity to inhibit pro-inflammatory cytokine production was much more potent than that of adenosine (Figure 2). Refractoriness of ANO to adenosine deaminase might be a plausible explanation for the potent anti-inflammatory activity of ANO (Figure 4B). However, anti-inflammatory activity of adenosine in the presence of EHNA was still inferior to that of ANO (Figure 4C and D), suggesting the existence of another functional difference between ANO and adenosine.

The intracellular signaling pathways induced by ANO and adenosine seem to differ. In LPS-stimulated peritoneal macrophages, ANO-mediated inhibition of TNF-α production was not reversed by H-89, whereas adenosine-mediated inhibition of TNF-α was completely recovered by H-89 (Figure 9). In accordance with our results, the non-selective AR agonist adenosine-5’-N-ethylcarboxamide (NECA) inhibited TNF-α production by LPS-stimulated murine macrophages via a signaling pathway that was independent of PKA and exchange protein activated by cAMP (Epac) [44].

Another cAMP-mediated suppressive mechanism of LPS-induced pro-inflammatory cytokine production involves the transcription factor c-Fos. c-Fos protein, which is upregulated by cAMP and stabilized following phosphorylation by LPS-activated Ikkβ, physically binds to the p65 subunit of NF-κB. Through this binding, the recruitment of p65:p65 homodimer to the *TNF-α* promoter region is reduced, resulting in the suppression of TNF-α production [27]. Induction of *c-fos* mRNA was partially suppressed by the phosphoinositide-3 kinase (PI3K) inhibitor Wortmannin but not by the PI3K inhibitor LY294002, suggesting a role of a Wortmannin-sensitive kinase in the induction of *c-fos* mRNA [27]. The role of c-Fos as an anti-inflammatory transcription factor was reported previously [45]. Macrophages collected from Fos^−/−^ mice showed significantly enhanced production of pro-inflammatory cytokines compared with macrophages from wild-type control mice [45]. In our study, ANO-mediated inhibition of TNF-α production by LPS-stimulated macrophages was partially recovered by Wartmannin (Figure 9). Furthermore, we observed upregulation of *c-fos* mRNA and c-Fos protein expression (Figure 10). These results suggest that up-regulation of c-Fos is, at least in part, responsible for ANO-mediated suppression of TNF-α production.

However, it should be noted that cAMP-mediated suppression of IL-6 release was not complete and that recruitment of p65 to the IL-6 promoter region was not reduced but rather enhanced by cAMP treatment [27], suggesting the presence of another mechanism for ANO-induced suppression of IL-6 secretion. Experiments are ongoing to address this issue.

## Conclusions

ANO is an oxidized product of adenosine at the N1 position of the adenine base moiety. Here, we have shown that ANO is contained in RJ and that it possesses anti-inflammatory activities both *in vitro* and *in vivo*. ANO was refractory to adenosine deaminase-mediated conversion to inosine and exhibited potent anti-inflammatory activities through signaling pathways different from those of adenosine. ANO was superior to GK2 (a common anti-inflammatory drug) in suppressing broad spectrum inflammatory mediators. Reflecting the effective anti-inflammatory effects *in vitro*, intravenous administration of ANO reduced lethality to LPS-induced endotoxin shock. Significant increases in the survival rate were also observed by oral administration of ANO, although the most efficacious ANO dosage needs to be addressed. These results suggest that ANO presents a potential strategy for the treatment of inflammatory disorders.

## Abbreviations

ANO: Adenosine N1-oxide
AR: Adenosine receptor
GK2: Dipotassium glycyrrhizate
CCPA: 2-Chloro-N^6^-cyclopentyladenosine
CGS21680: 2-[*p*-(2-Carboxyethyl)phenethylamino]-5’-N-ethylcarboxamideadenosine hydrochloride
IB-MECA: N^6^-(3-Iodobenzyl)adenosine-5’-N-methyluronamide
AMP: Adenosine monophosphate
cAMP: cyclic AMP
RJ: Royal jelly
LPS: Lipopolysaccharide
IFN-γ: Interferon-γ
muIFN-γ: murine IFN-γ
huIFN-γ: human IFN-γ
TLR: Toll-like receptor
PGE2: Prostaglandin E2
PKA: Protein kinase A
PI3K: Phosphoinositide-3 kinase
